# Comparison of different anticoagulation strategies for renal replacement therapy in critically ill patients with COVID-19: a cohort study

**DOI:** 10.1186/s12882-020-02150-8

**Published:** 2020-11-16

**Authors:** Frederic Arnold, Lukas Westermann, Siegbert Rieg, Elke Neumann-Haefelin, Paul Marc Biever, Gerd Walz, Johannes Kalbhenn, Yakup Tanriver

**Affiliations:** 1 Department of Medicine IV: Nephrology and Primary Care, Medical Center – University of Freiburg, Faculty of Medicine, University of Freiburg, Freiburg, Germany; 2 Institute of Medical Microbiology and Hygiene, Faculty of Medicine, University of Freiburg, Freiburg, Germany; 3 Berta-Ottenstein-Programme for Clinician Scientists, Faculty of Medicine, University of Freiburg, Freiburg, Germany; 4 Department of Medicine II: Division of Infectious Diseases, Medical Center – University of Freiburg, Faculty of Medicine, University of Freiburg, Freiburg, Germany; 5 Department of Medicine III: Interdisciplinary Medical Intensive Care, Medical Center – University of Freiburg, Faculty of Medicine, University of Freiburg, Freiburg, Germany; 6 Department of Cardiology and Angiology I, Heart Center, University of Freiburg, Freiburg, Germany; 7 Department of Anesthesiology and Critical Care, Medical Center – University of Freiburg, Faculty of Medicine, University of Freiburg, Freiburg, Germany

**Keywords:** COVID-19, SARS-CoV-2, Acute kidney injury, Renal replacement therapy, Anticoagulation, Critical care, Emerging communicable diseases

## Abstract

**Background:**

Critically ill coronavirus disease 2019 (COVID-19) patients have a high risk of acute kidney injury (AKI) that requires renal replacement therapy (RRT). A state of hypercoagulability reduces circuit life spans. To maintain circuit patency and therapeutic efficiency, an optimized anticoagulation strategy is needed. This study investigates whether alternative anticoagulation strategies for RRT during COVID-19 are superior to administration of unfractionated heparin (UFH).

**Methods:**

Retrospective cohort study on 71 critically ill COVID-19 patients (≥18 years), admitted to intensive care units at a tertiary health care facility in the southwestern part of Germany between February 26 and May 21, 2020. We collected data on the disease course, AKI, RRT, and thromboembolic events. Four different anticoagulatory regimens were administered. Anticoagulation during continuous veno-venous hemodialysis (CVVHD) was performed with UFH or citrate. Anticoagulation during sustained low-efficiency daily dialysis (SLEDD) was performed with UFH, argatroban, or low molecular weight heparin (LMWH). Primary outcome is the effect of the anticoagulation regimen on mean treatment times of RRT.

**Results:**

In patients receiving CVVHD, mean treatment time in the UFH group was 21.3 h (SEM: ±5.6 h), in the citrate group 45.6 h (SEM: ±2.7 h). Citrate anticoagulation significantly prolonged treatment times by 24.4 h (*P* = .001). In patients receiving SLEDD, mean treatment time with UFH was 8.1 h (SEM: ±1.3 h), with argatroban 8.0 h (SEM: ±0.9 h), and with LMWH 11.8 h (SEM: ±0.5 h). LMWH significantly prolonged treatment times by 3.7 h (*P* = .008) and 3.8 h (*P* = .002), respectively.

**Conclusions:**

UFH fails to prevent early clotting events in the dialysis circuit during COVID-19. For patients, who do not require effective systemic anticoagulation, regional citrate dialysis is the most effective strategy. For patients, who require effective systemic anticoagulation, the usage of LMWH results in the longest circuit life spans. The proposed anticoagulatory strategies are safe, can easily be monitored, and allow an individualized treatment.

**Graphical abstract:**

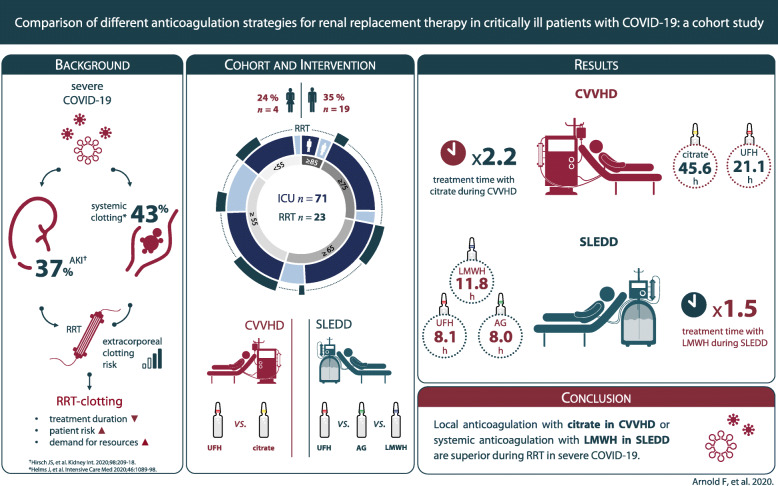

**Supplementary Information:**

The online version contains supplementary material available at 10.1186/s12882-020-02150-8.

## Background

The pandemic spread of severe acute respiratory syndrome coronavirus 2 (SARS-CoV-2) is challenging health care systems around the world as it carries significant morbidity and mortality [1]. Despite the predominance of respiratory symptoms, evidence emerges that coronavirus disease 2019 (COVID-19) is a multiorgan disease. SARS-CoV-2 copies can be detected in the kidneys, liver, and brain on autopsy [2]. Acute kidney injury (AKI) was registered in up to 37% of patients hospitalized for COVID-19 [3]. Endothelial and renal tropism of SARS-CoV-2 might contribute to this high incidence of AKI. Additionally, COVID-19 patients on intensive care unit (ICU) often develop a hyperinflammatory response with high levels of C-reactive protein (CRP) and excessive production of inflammatory cytokines [4]. This detrimental host response leads to sepsis and eventually septic shock, which will further aggravate renal function. Hence, current reports are indicating that direct and indirect effects of SARS-CoV-2 infection can lead to AKI in COVID-19 disease.

The high incidence of AKI in critically ill COVID-19 patients has led to regional shortages of dialysis supply, both in equipment and trained staff. In addition to an unusual high demand for hemodialysis, renal replacement therapy (RRT) has proven especially difficult due to hypercoagulability that results in intra- and extracorporeal clotting events in SARS-CoV-2 associated disease [5]. Clotting in the extracorporeal circulation and the dialysis filter frequently requires early termination of the treatment. This reduces gross quality of the dialysis, can lead to metabolic imbalances and fluid overload, increases blood loss due to more frequent changes of the system, and is likely to promote other complications for the patient. Hence, intensivists and nephrologists have acknowledged the urgent need for specific anticoagulation regimens in patients with COVID-19 requiring RRT [6, 7].

As a standard of care, critically ill patients with AKI, who require RRT, are treated either with continuous veno-venous hemodialysis (CVVHD) or with sustained low-efficiency daily dialysis (SLEDD). Systemic or local anticoagulation prevents tubing and dialyzers from clotting and is therefore necessary for effective delivery of RRT. Anticoagulation during dialysis is usually maintained with unfractionated heparin (UFH), which is the most commonly used anticoagulant worldwide for RRT [8]. Alternative methods of regional and systemic anticoagulation, including citrate, low molecular weight heparin (LMWH, e.g. enoxaparin), heparinoids (e.g. danaparoid), thrombin antagonists (hirudin and argatroban) or platelet inhibiting agents (prostacyclin and nafamostat) have been used successfully in the past [9]. However, their suitability in COVID-19-affected patients who require RRT have not been investigated so far.

Here, we report our experience with the use of regional citrate, LMWH, and argatroban after we encountered repetitive clotting events in extracorporeal circuits of COVID-19 patients on ICU, who were previously treated with UFH. The primary goal of this study was therefore to identify the most efficient anticoagulation strategies for RRT in a representative cohort of critically ill COVID-19 patients.

## Methods

In this single-center retrospective cohort study, we report data from the University of Freiburg Medical Center, Freiburg, Germany. Patients eligible for inclusion were adults (aged 18 years or older) who were treated at the Medical Center with a laboratory-confirmed SARS-CoV-2 infection between February 26 and May 21, 2020.

Demographic data, past medical history, clinical findings, laboratory values, treatment details, and outcome data of patients were extracted from electronic patient records by the investigators of the study (FA and LW). Follow-up data collection was continued until June 5, 2020.

For comparison, demographic, diagnostic data, and outcomes of all patients (aged 18 years or older) diagnosed with influenza virus infection and admitted to an intensive care unit at the University of Freiburg Medical Center between January 1, 2015 and May 21, 2020 were extracted from electronic patient records by the investigators of the study (FA and LW). All data were reviewed and verified by two physicians (FA and LW). Any uncertain records were not included in the final data analysis.

This study is in conformity with the ethical principles for medical research involving human subjects as laid down in the Helsinki Declaration (1964) and its amendments. Analysis and publication of the data was approved by the local ethics committee (155/20 to SR and 1016/20 to JK).

### Laboratory procedures

Laboratory confirmation of SARS-CoV-2 infection was performed with real-time RT-PCR methods from throat-swab samples. Concentrations of creatinine, CRP, procalcitonin (PCT), interleukin 6 (IL6), ferritin, D-dimer, antithrombin (AT), and fibrinogen were assessed in serum samples and detected during hospitalization. Activated partial thromboplastin time (aPTT) and anti-factor Xa activity were measured for monitoring anticoagulatory therapy. Frequency of examinations was determined by the treating physician. AKI and chronic kidney disease were diagnosed according to the respective KDIGO clinical practice guidelines [10, 11].

### Hemodialysis treatments and anticoagulation

All CVVHD treatments were performed with the multiFiltrate® or the multiFiltratePRO® system (both Fresenius Medical Care GmbH, Bad Homburg, Germany) using suitable dialysate solutions, CRRT dialyzers, and tubing kits. Regional citrate anticoagulation was maintained with the integrated multiFiltrate Ci-Ca® module. SLEDD treatments were performed with the GENIUS®90 system (Fresenius Medical Care GmbH, Bad Homburg, Germany) using suitable tubing kits and dialyzers for 10–12 h. Dialysate solutions were individually prepared at site. Dialysis parameters such as blood flow, dialysate flow, and ultrafiltration rate were set according to system based standards and adjusted at the discretion of the treating physician.

COVID-19 patients were treated on four different ICUs at the study center, providing a cumulative ICU capacity of 130 beds. Every ICU is operated by an independent department (medicine, anesthesiology, cardiovascular surgery, and general surgery) and patients were randomly assigned to the different ICUs.

Therapeutic anticoagulation with UFH and argatroban were maintained by permanent intravenous infusion of the substances. Effective anticoagulation with UFH was anticipated with an activated partial thromboplastin time (aPTT) of 1.5–2.5 times the baseline, for argatroban with an aPTT of 1.5–3.0 times the baseline. Regional anticoagulation with citrate was monitored by measuring post-filter ionized Ca^2+^ levels at least every 6 h. The infusion of citrate was titrated to achieve an ionized Ca^2+^ level < 0.35 mmol/L. Therapeutic anticoagulation with LMWH was maintained by subcutaneous injection of enoxaparin in a bodyweight adapted individual dose twice a day. Sufficient anticoagulation was supposed with an anti-factor-Xa activity of 0.5–1.0 IU/mL (measured 4 h after injection). Before each SLEDD treatment patients received an additional intravenous bolus of 1000 I.U. enoxaparin to account for clearance during SLEDD. This protocol has been published recently [12]. It reports data of three patients also included in this study. Prior to the COVID-19 pandemic established and routinely used anticoagulation protocols at the University Medical Center, included systemic anticoagulation with UFH for CVVHD and SLEDD and regional anticoagulation with citrate for CVVHD. Argatroban was administered in rare cases of UFH contraindication while systemic anticoagulation was required.

### Statistical analysis

Continuous and categorical variables were presented as median (IQR) and n (%), respectively. Either Mann-Whitney-U- or Kruskal-Wallis-Test were performed to compare medians. Chi-square test was performed to compare frequencies. Treatment times were calculated as mean duration per treatment (SEM or as specified in the figure legend). Either two-sided student’s *t*-test or analysis of variance (ANOVA) with Tukey’s multiple comparisons test were applied to compare treatment times and calculate *P-*values. A two-sided α of less than 0.05 was considered statistically significant. All statistical analyses were performed using Prism (Version 8.0.2), GraphPad Software, San Diego, California.

## Results

Between February 26 and May 21, 2020 a total of 203 patients with COVID-19 were admitted to the University Medical Center Freiburg, of which 48 patients (24%) died. During this regional outbreak, which followed the overall pandemic development in Germany, up to 96 patients with COVID-19 had to be treated simultaneously at the University Medical Center (Supplement, Fig. S1A). Of the 203 patients 124 were male (61%). Male patients were on average younger (62 vs 68 yrs) and had a more severe course of disease as indicated by ICU admission (44% vs 22%) or necessity for RRT (15% vs 5%) (Supplement, Fig. S1B). A complete overview of the demographic and clinical characteristics of patients on ICU is shown in Table 1.

**Table 1:** Baseline characteristics of the COVID-19 ICU-cohort

	RRT-cohort	Non-RRT-cohort
	***n =*** 23	***n*** = 48
Age		
Median (range), y	62 (38–79)	70 (44–92)
≥ 65 y	9 (39)	30 (63)
Female	4 (17)	13 (27)
Admission diagnosis COVID-19	18 (78)	37 (77)
Non-renal comorbidities^a^		
Any	20 (95)	38 (79)
Asthma/COPD	2 (10)	3 (6)
Atrial Fibrillation	3 (14)	12 (25)
Coronary Artery Disease	3 (14)	10 (21)
Hypertension	12 (57)	19 (40)
Malignancy	4 (19)	7 (15)
Diabetes mellitus	4 (19)	10 (21)
Obesity	3 (14)	3 (6)
Renal		
CKD ≥ G2^b^	2 (12)	7 (15)
Acute Kidney injury ≥ Stage 1	23 (100)	30 (63)
Creatinine, baseline^b^, mg/dL	1.00 (0.80–1.14)	0.90 (0.70–1.10)
Creatinine max, mg/dL	5.99 (3.32–7.93)	1.52 (1.04–2.98)
SARS-CoV-2-PCR to RRT-initiation, d	9 (0–39)	
Mechanical cardiorespiratory support		
Invasive mechanical ventilation	22 (96)	30 (63)
Vasopressor administration	23 (100)	34 (71)
Extracorporeal membrane oxygenation	10 (43)	11 (23)
Inflammation markers, max.		
C-reactive protein, mg/L (NR: < 5)^c^	311 (223–394)	240 (170–331)
Procalcitonin, μg/L (NR: < 0.05)	18.80 (8.70–43.80)	0.99 (0.38–3.49)
Interleukin 6, ng/L (NR: < 7)^d^	1889 (710–14,403)	320 (112–848)
Ferritin, μg/L (NR: 30–400)^e^	2565 (1493–3880)	1612 (807–2719)
COVID-19 targeted therapy		
any	20 (87)	40 (83)
Hydroxychloroquine	19 (83)	36 (75)
Lopinavir, Ritonavir	13 (57)	22 (46)
Remdesivir	0 (0)	1 (2)
Tocilizumab (Anti-IL6)	3 (13)	3 (6)
Cytokine filter (Cytosorb®)	1 (4)	1 (2)
Death	12 (52)	22 (46)

Data reported as counts (percentages) or median (interquartile range) unless otherwise indicated. NR: normal range. If not otherwise specified, data represent total cohorts (*n =* 23 / *n =* 48). Creatinine max. in RRT-cohort indicates highest value prior to initiation of hemodialysis. ^a^Data available from *n =* 21 / *n =* 48 individuals in RRT- / Non-RRT-cohort; ^b^*n =* 17 / *n =* 48, SI conversion factor to μmol/L, multiply by 88.4; ^c^*n =* 22 / *n =* 47; ^d^*n =* 22 / *n =* 46; ^e^*n =* 19 / *n =* 42

**Fig. 1:**
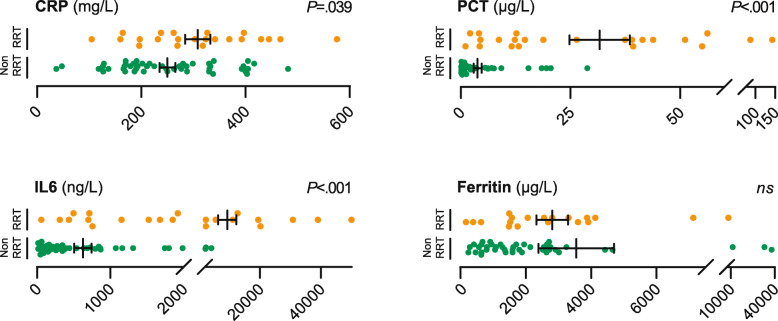
Distribution of inflammation markers in COVID-19 RRT- and Non-RRT-cohort. Dot plots show levels of selected inflammation markers CRP, PCT, IL6, and Ferritin. Orange dots () represent individual patients who underwent CVVHD or SLEDD. Green dots () represent patients who were not treated with RRT. Bars depict mean and standard error of the mean (SEM). *P-*values were calculated using a two-tailed student’s *t*-test. *ns*: not significant with α = .05. CRP available from *n =* 22 / *n =* 47 (RRT- / Non-RRT-cohort); PCT *n =* 23 / *n =* 48; IL6 *n =* 22 / *n =* 46; Ferritin *n =* 19 / *n* *=* 42

Seventy-one patients (35%) were treated on ICU. Of the ICU-cohort, 53 (75%) individuals developed AKI and 23 (32%) individuals were treated with RRT. Patients requiring RRT (RRT-cohort) were on average 8 years younger than patients in the Non-RRT-cohort (62 vs 70 yrs). We also observed a male predominance in our RRT-cohort and most patients that required RRT had higher rates of pre-existing comorbidities, such as chronic obstructive pulmonary disease, hypertension, and obesity. Of note, baseline renal function was not different between the two groups and most patients had no renal impairment before hospital admission. In line with their disease course, cardiorespiratory support (i.e. invasive mechanical ventilation, ECMO, vasopressors) was more often administered in the RRT-cohort. These patients showed significantly higher levels of the inflammation markers CRP, PCT, and IL6 (Fig. 1). Application of COVID-19 targeted therapies did not correlate with the requirement for RRT. Finally, there was a higher mortality rate in the RRT-cohort (52%) when compared to the Non-RRT-cohort (46%) (Table 1).

We were surprised by the high incidence of AKI, which frequently required RRT in critically ill patients diagnosed with COVID-19. The question emerged whether critically ill patients with other systemic viral infections had a similar incidence of renal complications at our hospital. Consequently, we compared the current COVID-19 ICU-cohort to a previous cohort of 200 influenza patients (including influenza A, B) treated on ICU at the University of Freiburg Medical Center from 2015 to 2020 (Supplement, Table S1). These cases represent all patients diagnosed with influenza and admitted to the ICU (13% of total influenza cases) during this 5 year period.

We were able to uncover that COVID-19 patients in fact have a higher demand for RRT than influenza patients (RRT-rates: 32% vs 19%, *P* = .020). This might indicate a more severe course of disease in COVID-19, which was further corroborated by the higher mortality rate of patients suffering from COVID-19 compared to influenza patients (48% vs 24%, *P* < .001). The observed male predominance in COVID-19 patients admitted to ICU was less pronounced in influenza patients (sex ratios: 1:3.2 vs 1:1.6). Overall, males required RRT more often than females during systemic viral infection, but especially when severe AKI was COVID-19 associated (Supplement, Fig. S2).

Initially, intravenous UFH was administered for systemic anticoagulation in patients who received RRT for COVID-19 related AKI. RRT was either performed as CVVHD or SLEDD. In these patients, we observed frequent clotting events in the extracorporeal circulation and the dialysis filter. Our data also indicated a higher rate of systemic thromboembolic events in the RRT-cohort (35% vs 19%) (Supplement, Table S2). Supporting this finding of hypercoagulability, we also noticed a trend towards higher levels of D-dimer (8.68 mg/L vs 5.01 mg/L, SI conversion factor to nmol/L: 5.476) as well as lower levels of fibrinogen (288 mg/dL vs 356 mg/dL) and AT (70% vs 76%) in the RRT-cohort (Supplement, Table S2 and Fig. S3). Subgroup analysis did not reveal any statistically different levels of prothrombotic markers between the different anticoagulatory regimens.

Due to frequent extracorporeal clotting events with early termination of treatments, anticoagulatory regimens were changed at the discretion of the treating physician according to the patient’s individual risk profile and departmental experience. As a result, patients requiring CVVHD were regularly treated with a regional anticoagulatory regimen with citrate. In cases where systemic anticoagulation was warranted, patients received either intravenous UFH or argatroban. Patients qualifying for SLEDD received either intravenous argatroban or subcutaneously administered LMWH as an alternative systemic anticoagulation regimen. Switches of individual treatment subgroups are depicted in Tables S3 and Table S4 in the Supplement. Importantly, patients’ baseline characteristics in subgroups according to dialysis and anticoagulatory regimen were comparable (Supplement, Table S3 and Table S4).

**Fig. 2:**
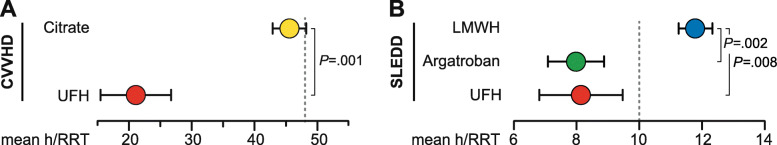
Treatment times of patients receiving CVVHD (**a**) or SLEDD (**b**). Circles show mean duration of treatment according to anticoagulant regimen. Error bars depict SEM. *P-*values were calculated using a two-tailed student’s *t*-test comparing the CVVHD-subgroups and ANOVA with Tukey’s multiple comparisons test comparing the SLEDD-subgroups. Dashed lines represent minimal expected treatment times (48 h for CVVHD, 10 h for SLEDD)

To investigate the efficiency of the different anticoagulatory regimens, we compared mean treatment times. In the context of CVVHD, 7 patients (treatments: *n* = 13) were administered UFH, 18 patients (*n* = 92) underwent regional anticoagulation with citrate. Mean treatment time in the citrate group was 45.6 h (SEM: ±2.7 h). Mean treatment time in the UFH group was 21.3 h (SEM: ±5.6 h). 81% of the measured aPTT values of the UFH subgroup and 98% of post-filter calcium levels were within the therapeutic range, indicating adequate dosing. Citrate anticoagulation prolonged treatment duration significantly by 24.4 h (*P* = .001), increasing mean treatment time more than twice. As a standard procedure time for CVVHD without change of dialysis filter or equipment, we defined 48 h. In the UFH group, just 8% of the treatments were running for at least 48 h. Compared to that, 45% of the treatments in the citrate group were running longer than 48 h (Table 2 and Fig. 2a).

**Table 2:** Counts, treatment times and therapeutic anticoagulation of CVVHD and SLEDD in COVID-19

	CVVHD		SLEDD		
	UFH	Citrate	UFH	Argatroban	LMWH
No. of RRT treatments	13	92	22	27	76
No. of patients	7	18	9	3	7
Total RRT duration, h	274.7	4192.6	179.1	215.6	896.4
Mean duration per RRT, h (SD)	21.1 (20.2)	45.6 (25.6)	8.1 (6.2)	8.0 (4.7)	11.8 (4.7)
Early RRT termination, %	92	55	64	67	30
Therapeutic range, %	81	98	100	96	78

Data reported as counts, mean values (standard deviation) and percentages. For the calculation of the percentage of early hemodialysis termination a minimum operating time per CVVHD treatment of at least 48 h was chosen. Percentages depict portion of treatments running less than 48 h. For the calculation of the percentage of early hemodialysis termination a minimum operating time per SLEDD treatment of at least 10 h was chosen. Percentages depict portion of treatments running less than 10 h. Percentage of aPTT-, post filter calcium, or anti-factor-Xa levels in therapeutic range (UFH: aPTT ≥45 s / 1.5 times baseline; citrate: ionized Ca^2+^ post filter < 0.35 mmol/L Argatroban: aPTT ≥45 s / 1.5 times baseline; LMWH ≥0.5 IU/mL)

Patients that underwent SLEDD procedures were administered systemic anticoagulation. Nine Patients (*n* = 22) received UFH, 3 patients (*n* = 27) argatroban, and 7 patients (*n* = 76) subcutaneously administered LMWH. 100% and 96% of aPTT levels in the UFH and argatroban group, respectively, as well as 78% of anti-factor-Xa levels were within the therapeutic range, indicating adequate dosing of respective anticoagulants (Table 2). Mean dialysis time in the UFH group was 8.1 h (SEM: ±1.3 h), in the argatroban group 8.0 h (SEM: ±0.9 h), and in the LMWH group 11.8 h (SEM: ±0.5 h). Use of LMWH significantly prolonged treatment times by 3.7 h (*P* = .008) and 3.8 h (*P* = .002) compared to the UFH group and argatroban group, respectively. Postulating a standard treatment time of 10 h for SLEDD procedures, 64% of treatments in the UFH group and 67% in the argatroban group had to be terminated early, whereas just 30% of treatments in the LMWH group were conducted for less than the anticipated 10 h. Hence, systemic anticoagulation with LMWH in COVID-19 patients receiving SLEDD results in a significant reduction of thromboembolic events in the extracorporeal circulation and permits sufficient dialysis in the majority of patients (Table 2 and Fig. 2b).

## Discussion

Regional outbreaks of SARS-CoV-2 pose huge challenges to health care providers since transmission dynamics lead to rapid and simultaneous admission of large numbers of moderate to critically ill patients. Mortality of critically ill patients is high compared to other pandemic viral diseases such as influenza (48% vs 24% in this study) [13–15]. Besides the early predominance of respiratory symptoms COVID-19 can quickly develop into a multiple organ dysfunction syndrome, which often involves the kidneys [2, 16]. As a result, critically ill patients suffering from COVID-19 more often required RRT than patients suffering from influenza (32% vs 19%).

This report depicts a single-center experience with a cohort of 71 critically ill COVID-19 patients and primarily aims to give useful practical advice to healthcare professionals during an ongoing global crisis and in view of potential future outbreaks. Due to high risk for AKI, SARS-CoV-2-outbreaks confront health care providers with an unusual high demand for hemodialysis infrastructure. Hypercoagulability in critically ill patients with COVID-19 complicates RRT by significantly reducing treatment times. Premature termination due to clotting events increases blood loss, causes electrolyte disturbances, promotes acid-base shifts, and complicates volume management [17–19]. Early termination of RRT increases workload and aggravates potential shortages in both staff and dialysis supplies [20]. Evidence-based anticoagulation strategies in this subgroup of COVID-19 patients ensuring efficient RRT are therefore urgently needed. With this study, we propose superior anticoagulation regimens for critically ill COVID-19 patients requiring RRT.

In our RRT-cohort, we observed unusually short treatment times for CVVHD and SLEDD in patients who were anticoagulated with UFH. First, 92% of CVVHD treatments had to be discontinued prematurely despite systemic anticoagulation with UFH. Second, administration of UFH in patients receiving SLEDD resulted in early treatment termination due to extracorporeal clotting in 64% of the cases. The anticoagulatory effect of UFH is conveyed by binding and potentiating the inhibitory actions of AT. The formation of an UFH-AT-complex inhibits mainly factor Xa and thrombin (factor IIa). Hence, reduced bioavailability of administered UFH, overwhelming activation of the coagulation cascade, as well as hereditary or acquired AT deficiency can all hamper the efficiency of UFH [21].

Severe COVID-19 is characterized by an excessive production of inflammatory proteins, as shown here and in other studies [4]. Several acute-phase proteins have been demonstrated to bind and inactivate UFH, thereby limiting its anticoagulatory effects [22]. Furthermore, we noticed a significant increase in prothrombotic proteins indicating hypercoagulability, which goes in line with the reported high incidence of thromboembolic events in COVID-19 patients [23]. High concentrations of prothrombotic factors could result in an increased fraction of functional factor Xa and thrombin, favoring cleavage of fibrinogen, and leading to clotting events. Ongoing coagulation and thrombus formation reduces circulating AT, which was most noticeable in our RRT-group. Lastly, age has been shown to be a prognostic marker for UFH resistance [24]. In the RRT-group, 39% of the patients were 65 years or older and therefore at risk for UFH resistance.

In patients with SLEDD, the LMWH enoxaparin significantly improved mean duration time in comparison to UFH and argatroban. There are factors which could contribute to superior anticoagulatory effects of LMWH. In contrast to UFH, application of LMWH does not lead to depletion of tissue factor pathway inhibitor (TFPI), which is a potent inhibitor of the extrinsic coagulation pathway that is triggered by tissue factor [25]. Additionally, LMWH has a longer half-life, better bioavailability, and lower affinity for plasma protein binding sites than UFH, which all contribute to a more reliable anticoagulation [9]. This is further corroborated by a recent study that demonstrated a survival benefit when COVID-19 patients with a high concentration of D-dimers or sepsis-induced coagulopathy were treated with LMWH [26].

Administration of citrate significantly improved mean treatment duration of CVVHD for COVID-19 patients. Regional anticoagulation with citrate is a suitable alternative to UFH in critically ill patients during CVVHD [27, 28]. In contrast to systemic anticoagulation, citrate complexes calcium. At a concentration of 4–6 mmol/L with an ionized calcium of < 0.2 mmol/L citrate prevents activation of both coagulation cascades and platelets. These widespread effects very well explain its superior ability to prevent extracorporeal clotting events. Since citrate provides regional anticoagulation, prevention of systemic thromboembolism with citrate is not feasible. However, regional anticoagulation with citrate and additional systemic anticoagulation tailored for patients with COVID-19 will further increase patient safety compared to sole systemic anticoagulation with UFH.

An increased risk for thromboembolism due to hypercoagulability with elevation of prothrombotic markers has frequently been observed and seems to be a hallmark of severe COVID-19 disease and poor outcome [5, 23, 29]. In our COVID-19 RRT-cohort, 35% of the patients developed intracorporeal thromboembolic events despite anticoagulation.

It is speculated that coagulopathy in COVID-19 patients might be caused by systemic inflammation [30]. However, recent studies also indicate a direct involvement of SARS-CoV-2 infection. Viral elements were detected in endothelial cells of multiple organs that might facilitate the induction of endotheliitis. COVID-19-endotheliitis could be a crucial factor for microvascular dysfunction [30, 31]. Significant increase of inflammation markers (e.g. CRP, PCT, IL6) is present in patients with SARS-CoV-2 infection at admission [32]. In our study, critically ill COVID-19 patients requiring RRT showed higher inflammation markers compared to those not requiring RRT. This could further aggravate hypercoagulability and predispose to intra- and extracorporeal clotting.

Limits of our study are a short observational period and the small sample size at a single-center. Since our study is retrospective in design and reflects clinical experience and decision-making during the first weeks of the COVID-19 pandemic in Germany, it was not possible to eliminate all confounders. Confounding variables may result from the switch of patients between different anticoagulatory regimens and different treatment group sizes. Subgroups treated with UFH are particularly small due to frequently observed complications and early adaption of alternative anticoagulation strategies. However, since UFH has been the standard choice of anticoagulation during RRT for decades empirical evidence in combination with our own study indicates that alternative anticoagulants might be preferable in COVID-19 associated hypercoagulability.

## Conclusions

In summary, our report adds to the growing body of evidence that COVID-19 can evolve into a multiorgan disease that frequently results in acute kidney injury with a high demand for RRT. Severe cases of COVID-19 are often accompanied by a state of hypercoagulability that poses a challenge to administer efficient RRT. Shortened lifespans of the dialysis circuit during renal replacement therapy in critically ill COVID-19 patients are a broadly recognized problem, yet very limited evidence exists about adequate anticoagulation strategies. Preliminary recommendations of single medical centers or health care authorities, that are mainly based on experts’ opinions are divergent, inconclusive, lack thorough data analysis, and therefore cannot be generalized.

We were able to demonstrate that mean RRT duration time can be improved significantly by using either regional anticoagulation with citrate for CVVHD or systemic anticoagulation with LMWH for SLEDD. However, the transfer of our data into clinical management is impeded by confounding factors. Despite its limitations, our findings should prompt further investigation and can be regarded as a foundation for future research to elucidate superior anticoagulation strategies for critically ill COVID-19 patients requiring RRT. Prolonged dialysis circuit lifespans have implications on patient safety and conserve critical resources. Ultimately, a better understanding of COVID-19 associated hypercoagulability is required to offer tailored anticoagulation strategies, to maintain extracorporeal circuit patency, and reduce overall mortality from thromboembolic events during this ongoing pandemic.

## Supplementary Information

**Additional file 1:** **Fig. S1.** Intensive care and renal replacement therapy in COVID-19 cases at the University of Freiburg Medical Center 02/26/2020–05/21/2020 (85 d). **Table S1.** COVID-19 and influenza patients treated on ICU. **Fig. S2.** RRT and mortality in critically ill COVID-19 and influenza patients. **Table S2.** Thromboembolic events and prothrombotic markers. **Fig. S3.** Distribution of prothrombotic markers in COVID-19 RRT- and Non-RRT-cohort. **Table S3.** Baseline characteristics according to anticoagulatory regimen during CVVHD. **Table S4.** Baseline characteristics according to anticoagulatory regimen during SLEDD

## Data Availability

The raw datasets analyzed in this study are available from the corresponding author on reasonable request after de-identification.

## Abbreviations

AKI: Acute kidney injury
aPTT: Activated partial thromboplastin time
AT: Antithrombin
COVID-19: Coronavirus disease 2019
CRP: C-reactive protein
CVVHD: Continuous veno-venous hemodialysis
ICU: Intensive care unit
TFPI: Tissue factor pathway inhibitor
IL6: Interleukin 6
IQR: Interquartile range.
LMWH: Low molecular weight heparin
PCT: Procalcitonin
RRT: Renal replacement therapy
SARS-CoV-2: Severe acute respiratory syndrome coronavirus 2
SEM: Standard error of the mean
SLEDD: Sustained low-efficiency daily dialysis
UFH: Unfractionated heparin
