# Supplementary material for: Comparison of different anticoagulation strategies for renal replacement therapy in critically ill patients with COVID-19: a cohort study
Source: BMC Nephrol. 2020 Nov 16;21:486. doi: 10.1186/s12882-020-02150-8 (PMC7668013; doi:10.1186/s12882-020-02150-8)
Supplement: Supplementary file 1 — Additional file 1: Fig. S1. Intensive care and renal replacement therapy in COVID-19 cases at the University of Freiburg Medical Center 02/26/2020–05/21/2020 (85 d). Table S1. COVID-19 and influenza patients treated on ICU. Fig. S2. RRT and mortality in critically ill COVID-19 and influenza patients. Table S2. Thromboembolic events and prothrombotic markers. Fig. S3. Distribution of prothrombotic markers in COVID-19 RRT- and Non-RRT-cohort. Table S3. Baseline characteristics according to anticoagulatory regimen during CVVHD. Table S4. Baseline characteristics according to anticoagulatory regimen during SLEDD [file 12882_2020_2150_MOESM1_ESM.pdf]

Supplemental Material

Comparison of different anticoagulation strategies for renal replacement therapy in critically ill patients with COVID-19: a cohort study

Frederic Arnold; Lukas Westermann; Siegbert Rieg; Elke Neumann-Haefelin; Paul Biever; Gerd Walz; Johannes Kalbhenn; Yakup Tanriver

Content

Figure S1 | Intensive care and renal replacement therapy in COVID-19 cases at the University of Freiburg Medical Center 02/26/2020–05/21/2020 (85 d)

Table S1 | COVID-19 and influenza patients treated on ICU

Figure S2 | RRT and mortality in critically ill COVID-19 and influenza patients

Table S2 | Thromboembolic events and prothrombotic markers

Figure S3 | Distribution of prothrombotic markers in COVID-19 RRT- and Non-RRT-cohort

Table S3 | Baseline characteristics according to anticoagulatory regimen during CVVHD

Table S4 | Baseline characteristics according to anticoagulatory regimen during SLEDD

Figure S1 | Intensive care and renal replacement therapy in COVID-19 cases at the University of Freiburg Medical Center 02/26/2020–05/21/2020 (85 d)

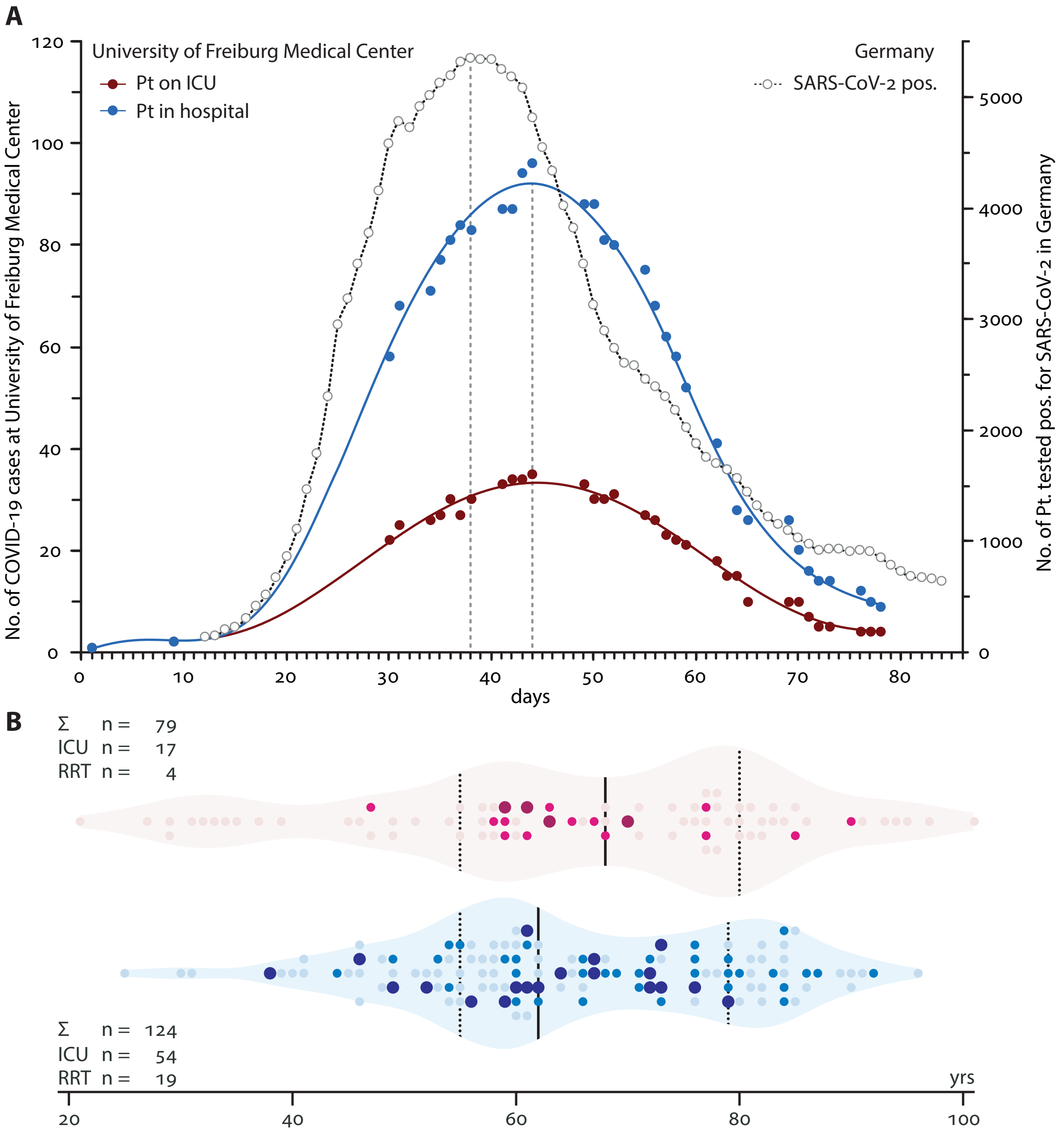

Figure S1 | (A) COVID-19 cases at the University of Freiburg Medical Center 02/26/2020–05/21/2020 (85 d). The blue graph (●) shows the total number of SARS-CoV-2 positive patients treated at the University Medical Center Freiburg at a given time point within a 85 day period (02/26/2020–05/21/2020) during the peak of the COVID-19 pandemic in Germany. The red graph (●) depicts the number of COVID-19 positive patients treated on an intensive care unit at University Medical Center. The dashed graph (○) shows the number of patients tested positive for SARS-CoV-2 in Germany. The temporal relation to the transmission dynamics and course of the pandemic in Germany with an approximate delay from positive testing to hospitalization of 5–7 days is demonstrated by the count of positive SARS-CoV-2 tests. (Data reported by Robert Koch Institute and local health authorities); **(B) Age distribution, ICU admission and RRT application in COVID-19-cohort.** Light dots depict all; dark dots depict individuals treated on an ICU for COVID-19. Enlarged dots mark individuals receiving RRT (● females; ● males). Median age (quartile ranges): females 68 yrs (55–80), males 62 yrs (55–79).

Table S1 | COVID-19 and influenza patients treated on ICU

	Σ (%)	all ages ♀	all ages ♂	≥85 yrs ♀	≥85 yrs ♂	75–84 yrs ♀	75–84 yrs ♂	65–74 yrs ♀	65–74 yrs ♂	55–64 yrs ♀	55–64 yrs ♂	<55 yrs ♀	<55 yrs ♂
**COVID-19**													
ICU admission	71 (100)	17	54	2	3	2	13	4	15	8	14	1	9
RRT on ICU	23 (32)*	4	19	0	0	0	2	1	6	3	7	0	4
Death on ICU	34 (48)#	8	26	1	3	2	7	1	9	4	5	0	2
**Influenza**													
ICU admission	200 (100)	78	122	4	5	15	19	19	32	18	34	22	32
RRT on ICU	38 (19)*	13	25	0	0	2	4	3	4	3	9	5	8
Death on ICU	48 (24)#	16	32	1	1	3	4	4	8	2	9	6	10

COVID-19 (02/26/2020–05/21/2020) and influenza patients (01/01/2015–05/21/2020) treated on ICU at the University of Freiburg Medical Center. The rates of RRT and Death on ICU were significantly higher in the COVID-19 cohort. **P*=.020 and #P<.001; *P*-values were calculated using Chi-square test.

Figure S2 | RRT and mortality in critically ill COVID-19 and influenza patients

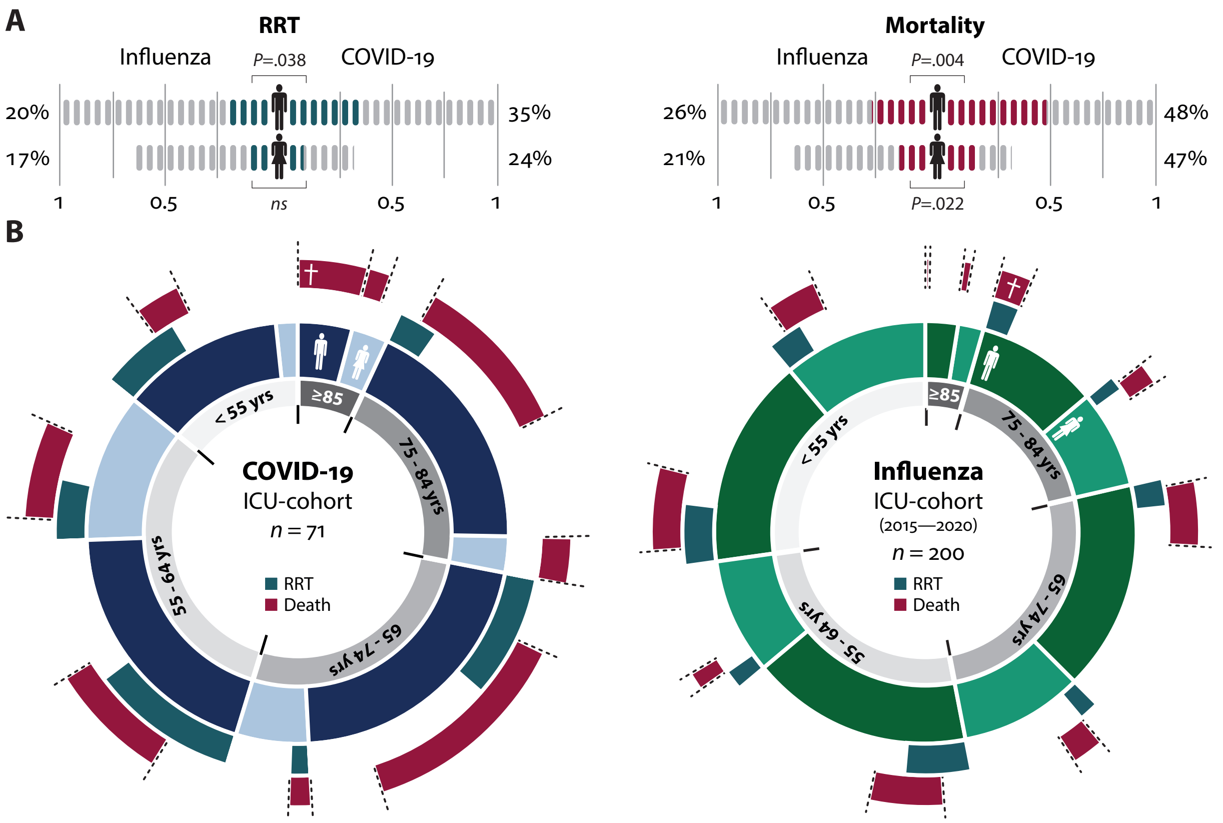

Figure S2 | RRT and mortality in critically ill COVID-19 and influenza patients. (A) Comparison of RRT application and mortality rates according to gender. Data from *n*=71 patients treated for COVID-19 on an ICU at the University of Freiburg Medical Center from 02/26/2020–05/21/2020 and from *n*=200 patients treated for influenza on an ICU at the University of Freiburg Medical Center in a five year period from 2015–2020. Graphs sized in proportion to sum of male cases. Mortality and RRT rates refer to gender specific counts. **(B)** Sunburst diagrams show RRT application and mortality-rate according to gender and age group for the same COVID-19 and influenza cohorts. Inner circles represent age groups; outer circles represent gender. Turquoise segments (■) depict RRT application. Red segments (■) depict deaths. *P*-values were calculated using Chi-square test.

Table S2 | Thromboembolic events and prothrombotic markers

	RRT-cohort *n*=23	Non-RRT-cohort *n*=48
Anticoagulation prior to hospitalization	5 (22)	11 (23)
Intracorporeal thromboembolic events	8 (35)	9 (19)
Prothrombotic markers		
D-dimer max, mg/L (NR: <0,5)[a]	8.68 (5.00–35.00)	5.01 (2.30–16.72)
Antithrombin min, % (NR: 79–120)[b]	70 (61–92)	76 (70–93)
Fibrinogen min, mg/dL (NR: 170–420)[c]	288 (230–404)	356 (158–564)
Fibrinogen max, mg/dL (NR: 170–420)[d]	580 (422–673)	559 (465–681)

Data reported as counts (percentages) or median (interquartile range). NR: normal range. For patients who underwent extracorporeal membrane oxygenation (ECMO) D-dimer max values were obtained prior to ECMO implantation. [a]Data available from *n=19* / *n=44* in RRT- / Non-RRT-cohort, SI conversion factor to nmol/L, multiply by 5.476; [b]*n*=15 / *n*=16; [c]*n*=15 / *n*=15; [d]*n*=19 / *n*=28.

Figure S3 | Distribution of prothrombotic markers in COVID-19 RRT- and Non-RRT-cohort

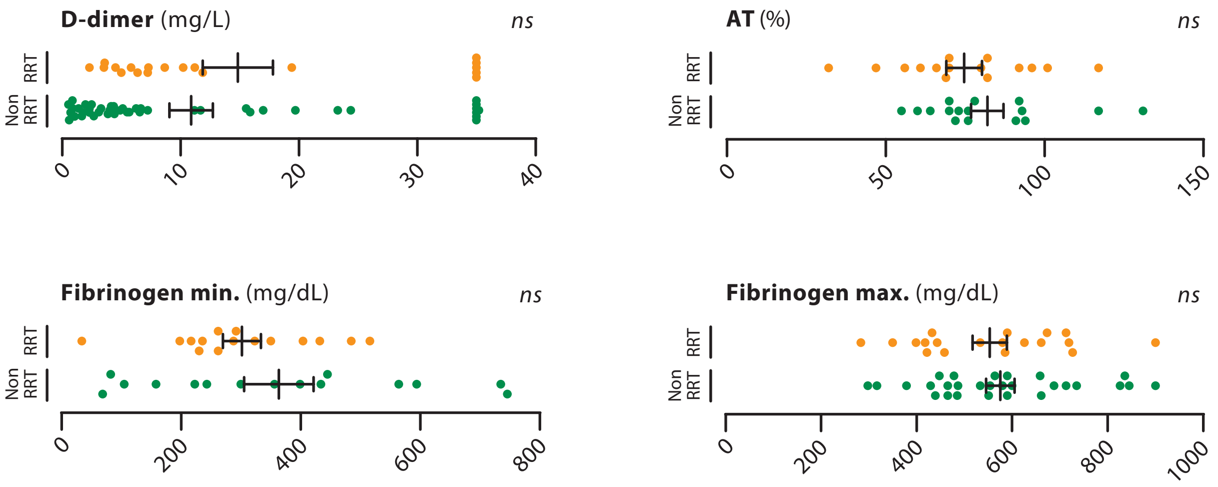

Figure S3 | Distribution of prothrombotic markers in COVID-19 RRT- and Non-RRT-cohort. Dot plots show levels of D-dimer, antithrombin (AT) and fibrinogen. Orange dots (●) represent individual patients who underwent CVVHD or SLEDD. Green dots (●) represent patients who were not treated with RRT. Bars depict mean and standard error of the mean (SEM). P-values were calculated using a two-tailed student's *t*-test. *ns*: not significant with α=.05. D-dimer available from *n*=19 / *n*=44 in RRT- / Non-RRT-cohort; AT *n*=15 / *n*=16; Fibrinogen min *n*=15 / *n*=15; Fibrinogen max *n*=19 / *n*=28.

Table S3 | Baseline characteristics according to anticoagulatory regimen during CVVHD

**CVVHD**	**UFH** *n*=7	**Citrate** *n*=18
Age		
Median (range), y	59 (49–73)	62 (38–79)
≥ 65 y	1 (14)	8 (44)
Female	0 (0)	3 (17)
Admission diagnosis COVID-19	6 (86)	14 (78)
Non-renal comorbidities[a]		
Any	6 (86)	15 (94)
Asthma/COPD	1 (14)	2 (13)
Atrial Fibrillation	0 (0)	2 (13)
Coronary Artery Disease	1 (14)	3 (19)
Hypertension	5 (71)	10 (63)
Malignancy	2 (29)	3 (19)
Diabetes mellitus	1 (14)	3 (19)
Obesity	1 (14)	3 (19)
Renal		
CKD ≥ G2[b]	0 (0)	2 (12)
Acute Kidney injury ≥ Stage 1	7 (100)	18 (100)
Creatinine, baseline[b], mg/dL	1.00 (0.90–1.18)	1.00 (0.78–1.21)
Mechanical cardiorespiratory support		
Invasive mechanical ventilation	7 (100)	18 (100)
Vasopressor administration	7 (100)	18 (100)
ECMO	1 (14)	8 (44)
Inflammation markers, max.		
C-reactive protein, mg/L (NR: <5)[c]	370 (238–430)	318 (235–395)
Procalcitonin, µg/L (NR: <0.05)	39.30 (4.32–56.20)	22.60 (7.64–42.00)
Interleukin 6, ng/L (NR: <7)[d]	1679 (700–1923)	2285 (736–19810)
Ferritin, µg/L (NR: 30–400)[e]	2446 (1216–5022)	2064 (1489–3598)
COVID-19 targeted therapy		
any	7 (100)	17 (94)
Hydroxychloroquine	7 (100)	16 (89)
Lopinavir, Ritonavir	6 (86)	10 (56)
Remdesivir	0 (0)	0 (0)
Tocilizumab (Anti-IL6)	0 (0)	3 (17)
Cytokine filter (Cytosorb ®)	0 (0)	1 (6)
Intracorporeal thromboembolic events	1 (14)	2 (11)
Anticoagulation prior to RRT	1 (14)	3 (17)
Bleeding events	0 (0)	1 (6)
Death	1 (14)	10 (56)
Switch to other CVVHD regimen	2 (29)	1 (6)
Switch to any other RRT regimen	7 (100)*	5 (28)*

Data reported as counts (percentages) or median (interquartile range) unless otherwise indicated. NR: normal range. If not otherwise specified, data represent total cohorts (*n*=7 / *n*=18) and differences were not significant between groups. [a]Data available from *n*=7 / *n*=16 individuals in UFH- / Citrate-cohort; [b]*n*=7 / *n*=14, SI conversion factor to µmol/L, multiply by 88.4; [c]*n*=7 / *n*=17 ; [d]*n*=7 / *n*=17; [e]*n*=6 / *n*=15. *Asterisk depicts significant difference between groups (*P*=.001, *P*-value calculated using Chi-square test).

Table S4 | Baseline characteristics according to anticoagulatory regimen during SLEDD

SLEDD	UFH *n*=9	Argatroban *n*=3	LMWH *n*=7
Age			
Median (range), y	62 (46–76)	59 (53–73)	56 (46-73)
≥ 65 y	3 (33)	1 (33)	2 (29)
Female	1 (11)	0 (0)	0 (0)
Admission diagnosis COVID-19	6 (67)	2 (67)	6 (86)
Non-renal comorbidities[a]			
Any	7 (88)	3 (100)	5 (71)
Asthma/COPD	2 (25)	1 (33)	1 (17)
Atrial Fibrillation	0 (0)	0 (0)	1 (17)
Coronary Artery Disease	2 (25)	1 (33)	0 (0)
Hypertension	4 (50)	2 (67)	3 (50)
Malignancy	2 (25)	1 (33)	1 (17)
Diabetes mellitus	2 (25)	1 (33)	1 (17)
Obesity	1 (13)	1 (33)	1 (17)
Renal			
CKD ≥ G2[b]	0 (0)	0 (0)	0 (0)
Acute Kidney injury ≥ Stage 1	9 (100)	3 (100)	6 (100)
Creatinine, baseline[b], mg/dL	1.00 (0.80–1.10)	1.10 (1.00–1.18)	0.90 (0.85–1.05)
Mechanical cardiorespiratory support			
Invasive mechanical ventilation	8 (89)	3 (100)	7 (100)
Vasopressor administration	9 (100)	3 (100)	7 (100)
ECMO	3 (33)	1 (33)	3 (43)
Inflammation markers, max.			
C-reactive protein, mg/L (NR: <5)[c]	360 (307–441)	393 (196–430)	381 (313–434)
Procalcitonin, µg/L (NR: <0.05)	26.40 (12.40–40.35)	26.40 (2.11–41.40)	39.30 (18.80–56.20)
Interleukin 6, ng/L (NR: <7)[c]	955 (450–4566)	700 (499–1854)	1504 (451–2810)
Ferritin, µg/L (NR: 30–400)[c]	3300 (1870.5–6378)	1489 (396–1580)	2446 (1219–5022)
COVID-19 targeted therapy			
any	8 (89)	3 (100)	5 (71)
Hydroxychloroquine	8 (89)	3 (100)	5 (71)
Lopinavir, Ritonavir	6 (67)	3 (100)	4 (57)
Remdesivir	0 (0)	0 (0)	0 (0)
Tocilizumab (Anti-IL6)	0 (0)	0 (0)	0 (0)
Cytokine filter (Cytosorb ®)	0 (0)	0 (0)	0 (0)
Intracorporeal thromboembolic events	0 (0)	0 (0)	0 (0)
Anticoagulation prior to RRT	2 (22)	1 (33)	2 (29)
Bleeding events[d]	1 (11)	0 (0)	1 (14)
Death	3 (33)	0 (0)	2 (29)
Switch to other SLEDD regimen	1 (11)	1 (37)	0 (0)
Switch to any other RRT regimen	7 (78)	2 (67)	2 (29)

Data reported as counts (percentages) or median (interquartile range) unless otherwise indicated. NR: normal range. If not otherwise specified, data represent total cohorts (*n*=9 / *n*=3 / *n*=7) and differences were not significant between groups. [a]Data available from *n*=9 / *n=3* / *n=5* individuals in UFH- / Argatroban- / LMWH-cohort; [b]*n*=8 / *n*=3 / *n*=4, SI conversion factor to µmol/L, multiply by 88.4; [c]*n*=9 / *n=3* / *n=5*. [d]Postoperative bleeding event occurring under different anticoagulatory regimen in a single patient.
